# Lateralized overgrowth with vascular malformation caused by a somatic *PTPN11* pathogenic variant: Another piece added to the puzzle of mosaic RASopathies

**DOI:** 10.1002/gcc.23086

**Published:** 2022-07-16

**Authors:** Alessandro Mussa, Antonella Turchiano, Simona Cardaropoli, Paola Coppo, Antonino Pantaleo, Rosanna Bagnulo, Carlotta Ranieri, Matteo Iacoviello, Antonella Garganese, Alessandro Stella, Stefano Gabriele Vallero, Daniele Bertin, Federica Santoro, Diana Carli, Giovanni Battista Ferrero, Nicoletta Resta

**Affiliations:** ^1^ Department of Public Health and Pediatric Sciences University of Torino Torino Italy; ^2^ Pediatric Clinical Genetics Unit Regina Margherita Children's Hospital, Città della Salute e della Scienza Torino Italy; ^3^ Division of Medical Genetics, Department of Biomedical Sciences and Human Oncology (DIMO) University of Bari “Aldo Moro” Bari Italy; ^4^ Pediatric Dermatology, Regina Margherita Children's Hospital Città della Salute e della Scienza di Torino Torino Italy; ^5^ Unit of Medical Genetics Ospedale Consorziale Policlinico di Bari Bari Italy; ^6^ Pediatric Onco‐Hematology, Regina Margherita Children's Hospital Città della Salute e della Scienza di Torino Torino Italy; ^7^ Pathology Unit, Department of Medical Sciences University of Torino Torino Italy; ^8^ Department of Clinical and Biological Sciences University of Torino Orbassano Italy

**Keywords:** astrocytoma, *FGFR1*, mosaicism, overgrowth, *PTPN11*, RASopathies

## Abstract

Lateralized/segmental overgrowth disorders (LOs) encompass a heterogeneous group of congenital conditions with excessive body tissue growth. Documented molecular alterations in LOs mostly consist of somatic variants in genes of the PI3KCA/AKT/mTOR pathway or of chromosome band 11p15.5 imprinted region anomalies. In some cases, somatic pathogenic variants in genes of the RAS/MAPK pathway have been reported. We present the first case of a somatic pathogenic variant (T507K) in *PTPN11* causing a LO phenotype characterized by severe lateralized overgrowth, vascular proliferation, and cerebral astrocytoma. The T507K variant was detected in DNA from overgrown tissue in a leg with capillary malformation. The astrocytoma tissue showed a higher *PTPN11* variant allele frequency. A pathogenic variant in *FGFR1* was also found in tumor tissue, representing a second hit on the RAS/MAPK pathway. These findings indicate that RAS/MAPK cascade overactivation can cause mosaic overgrowth phenotypes resembling PIK3CA‐related overgrowth disorders (PROS) with cancer predisposition and are consistent with the hypothesis that RAS/MAPK hyperactivation can be involved in the pathogenesis of astrocytoma. This observation raises the issue of cancer predisposition in patients with RAS/MAPK pathway gene variants and expands genotype spectrum of LOs and the treatment options for similar cases through inhibition of the RAS/MAPK oversignaling.

## 1 INTRODUCTION

Lateralized/segmental overgrowth (LO) is defined as an increase in tissue growth of various origins (skeletal, muscular, fibrous, vascular, adipose, or any association of these) in any region of the body.^1^ LOs are clinical entities increasingly defined at a molecular level thanks to high‐depth next generation sequencing (NGS) approaches.^2^Among LOs, PROS (*PIK3CA*‐related overgrowth spectrum) and BWSp (Beckwith–Wiedemann spectrum) represent the most common conditions.^3^, ^4^ However, nearly half of cases with a LO/PROS‐like phenotype do not harbor pathogenic variants in *PIK3CA*.^5^Somatic variants in other genes of the PI3K/AKT/mTOR signaling pathway (such as *PIK3R1*, *AKT1*, *PTEN*, and *MTOR)* or in the vascular proliferation cascade (such as *TEK*, *RASA1*, *GNAQ*, and *GNA11)* have been linked to overlapping overgrowth±vascular malformation phenotypes.^6^, ^7^ More recently, also somatic pathogenic variants in some of the genes of the RAS/mitogen‐activated protein kinase (RAS/MAPK) pathway (*HRAS*, *KRAS*) have been implicated in the pathogenesis of phenotypes with LO and vascular anomalies.^5^ Up to now, there have been no reports of somatic pathogenetic *PTPN11* variants in patients with LO and vascular abnormalities. *PTPN11* is the first discovered and the most frequently mutated genes of the RASopathies, a clinically defined group of overlapping syndromes caused by germline mutations in genes of components and regulators of the RAS/MAPK pathway including neurofibromatosis type 1, Noonan (NS) and Noonan with multiple lentigines, Costello, cardio‐facio‐cutaneous, and Legius syndromes.^8^ Mosaic variants in *PTPN11* are associated with isolated juvenile myelomonocytic leukemia (JMML, a neoplasm NS patients are prone to develop),^8^ as well as other types of cancers, including astrocytoma (COSMIC Database: Genomic Mutation ID: COSM13036). In our study, we describe the first case of a somatic pathogenic variant in *PTPN11* causing a congenital and progressive LO phenotype with vascular proliferation mimicking diffuse capillary malformation with overgrowth/Klippel–Trenaunay syndrome and characterized by development of cerebral astrocytoma in infancy. Our findings deepen the current knowledge of the LOs and mosaic RASopathies, providing insights into their pathogenesis and links to other somatic overgrowth disorders caused by perturbation of related gene signaling networks, such as the PI3K/AKT/mTOR pathway.^9^

## 2 MATERIALS AND METHODS

The study was approved by the ethics committee of the Città della Salute e della Scienza University Hospital of Torino, Italy (IRB approval ID 86/2022‐35286, March 28, 2022). The patient and her parents provided written informed consent to the study. Genomic DNA was extracted from peripheral blood cells, skin biopsy, and salivary swabs using the QIAamp Mini Kit (Qiagen, Hilden, Germany), according to the manufacturer's instructions, and quantified on a BioSpectrometer Plus (Eppendorf, Hamburg, Germany). Genomic DNA extraction from formalin‐fixed paraffin‐embedded (FFPE) astrocytoma tissue was carried out by Maxwell® 16 FFPE Tissue LEV DNA Purification Kit (PROMEGA) according to the manufacturer's instructions. Targeted deep sequencing using a custom panel of 17 genes involved in the PI3K/AKT/mTOR pathway (*PIK3CA*, *PIK3R1*, *PIK3R2*, *TEK*, *TSC2*, *GNAQ*, *TSC1*, *MTOR*, *PTEN*, *AKT3*, *AKT2*, *DEPDC5*, *AKT1*, *CCND2*, *NPRL3*, *GNA11*, *RASA1*)^10^ was performed on DNA from the affected lower extremity skin biopsy. Since this first analysis returned normal results, we carried out targeted deep sequencing also on tumor, salivary swab, and peripheral blood DNA using a custom panel of 15 genes involved in the RASopathies (*A2ML1, BRAF, CBL, HRAS, KRAS, MAP2K1, MAP2K2, NF1, NRAS, PTPN11, RAF1, RT1, SHOC2, SOS1, SPRED1)*. The two custom panels were both designed online using the Design Studio tool provided by Illumina (designstudio.illumina.com) to analyze the CDSs (±25 bp of intronic flanking regions) of the selected genes. Libraries were prepared using AmpliSeq for Illumina Kit (Ilumina, San Diego, CA) according to the manufacturer's instructions. Sequencing runs were performed on an Illumina MiSeq instrument according to the manufacturer's instructions by using a standard flow and V2 300 cycle cartridge (Ilumina). The Illumina sequencing produced a mean of 2 039 911 reads with an average coverage depth of about 3715 per sample. The uniformity of base coverage was 99.95% for the first panel and 100% for the second one. Data analysis was performed by Local Run Manager and BaseSpace Variant Interpreter software, both provided by Illumina. Alignments were visually verified with Alamut Visual 2.13‐0. Online databases, including dbSNP (www.ncbi.nlm.nih.gov/snp), gnomAD (nomad.broadinstitute.org), and ClinVar (www.ncbi.nlm.nih.gov/clinvar), were used. Results were confirmed by direct sequencing using the BigDye Terminator v1.1 Cycle Sequencing Kit on SeqStudio Genetic Analyzer (Applied Biosystems) according to the manufacturer's instructions.

Further tumor DNA analysis was conducted by a next generation sequencing ‐ NGS hot spot panel of the following genes (exons): *KT1* (3), *ALK* (21–25), *BRAF* (11, 15), *CDK4* (2), *CDKN2A* (1*,2,3), *CTNNB1* (3), *DDR2* (17), *DICER1* (24, 25), *EGFR* (18–21), *ERBB2* (8, 17, 20), *ERBB4* (10, 12), *FBXW7* (7–11), *FGFR1* (12, 14), *FGFR2* (7, 12, 14), *FGFR3* (7, 9, 14, 16), *FOXL2* (1*), *GNA11* (4, 5), *GNAQ* (4, 5), *GNAS* (8), *H3F3A* (2*), *H3F3B* (2*), *HIST1H3B* (1), *HRAS* (2–4), *IDH1* (4), *IDH2* (4), *KIT* (8–11, 13, 17, 18), K*RAS* (2–4), *MAP2K1* (2, 3), *MET* (2, 14–20), *MYOD1* (1), *NRAS* (2–4), *PDGFRA* (12, 14, 18), *PIK3CA* (2*,3,6*,8,10,21), *PTPN11* (3), *RAC1* (3), *RAF1* (7,10,12,13*,14*,15*), *RET* (11, 13, 15, 16), *ROS1* (38*,41*), *SF3B1* (15–17), *SMAD4* (8–12), *TERT* (promoter*,1*,8*,9*,13*), and *TP53* (2–11). Variant pathogenicity was assessed according to the ACMG guidelines.^11^

## 3 RESULTS

### 3.1 Patient description

**FIGURE 1:**
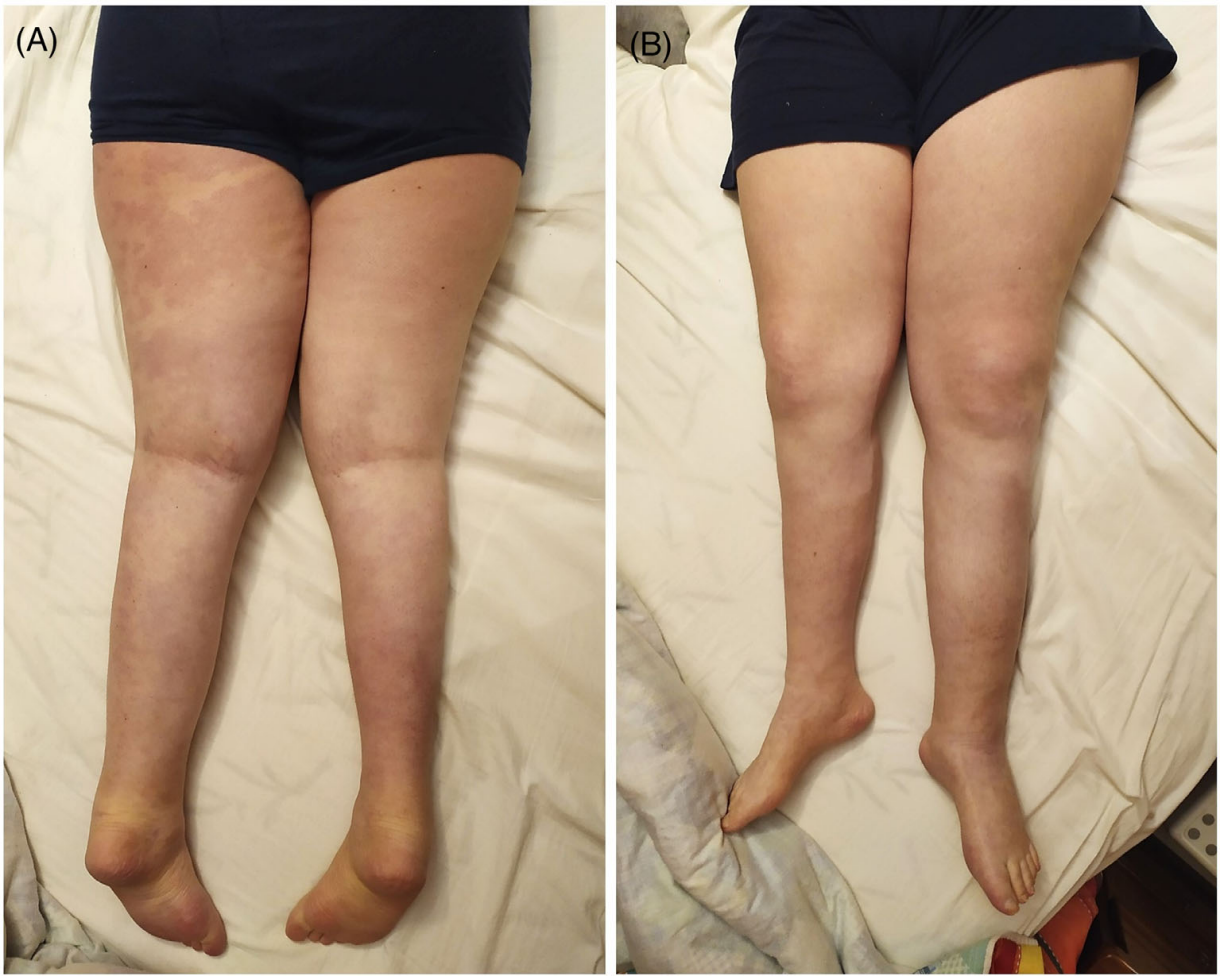
Lateralized overgrowth with vascular malformation in our patient. Note the asymmetry in leg length and girth and the plain vascular strain on the left leg

**FIGURE 2:**
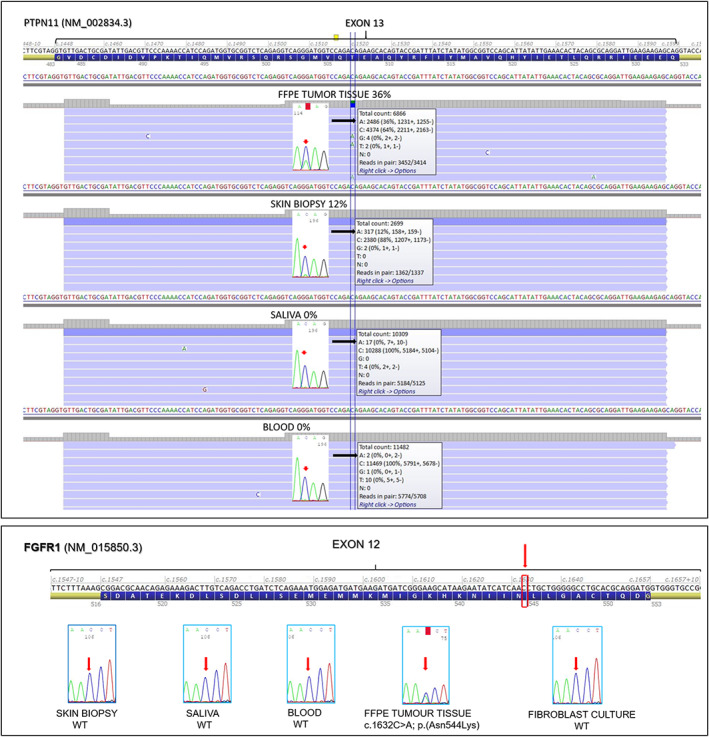
Biopsy of the hypotalamic mass showed a proliferation of monomorphic piloid cells in a uniform myxoid background (A, original magnification ×100). The neoplastic cells stained strongly with antibodies to GFAP (A, inset) and displayed a tendency to cluster around blood vessels (B, original magnification ×200)

The patient was born at 41 weeks of gestation from healthy and non‐consanguineous parents. Pregnancy was uneventful. Neonatal weight was 3520 g (+0.12 Standard Deviation Score [SDS]). At birth, she presented diffuse capillary vascular malformations with geographical margins involving upper and lower extremities, chest and face. She presented normal development during childhood and had no health issues. At 10 years of age, she was diagnosed with leg length discrepancy with left leg 2 cm longer than right leg and treated with a shoelift (Figure 1). Deep vein Doppler ultrasound and lower limb X‐rays were normal. At 13 years of age, she was admitted at the Regina Margherita Children's Hospital in Torino for hypothalamic intracranial hemorrhage. There was no evidence of cerebral vascular malformations on CT scan angiography. Brain MRI revealed a hypothalamic mass involving the optic chiasm. She underwent bilateral external ventricular drain placement, removed after 14 days, after the partial progressive spontaneous hemorrhage reabsorption. Hypothalamic mass biopsy histology revealed a pilomyxoid astrocytoma (Figure 2). She was initially treated with carboplatin and vincristine showing extreme toxicity reactions with peripheral neuropathy, severe nausea, and thrombocytopenia, so treatment was interrupted. She had also several comorbidities during treatment, such as pneumothorax, infections, hypernatremia, hypertension, dysautonomic dysfunction, lumbosacral anterolisthesis, anti‐D and anti‐C alloimmunization to transfusions. Last MRI showed stable disease. At cancer diagnosis she underwent a clinical genetics evaluation because clinical symptoms were suggestive of Klippel–Trenaunay syndrome. At clinical examination, she presented weight 74 kg (+2.02 SDS), height 175 cm (+2.62 SDS), OFC 57 cm (+2.67 SDS), consistent with familiar height target. No facial dysmorphisms were noted. She had a large capillary hemangioma covering a sizeable region in the body left side and involving leg, back, abdomen, and arm. In addition, the left leg was longer and larger compared to the right one. No anomalies were found at Doppler ultrasound of the lower limbs. A skin biopsy of the angioma of the overgrown leg was performed, concurrently with blood and buccal swab samplings for DNA analysis.

### 3.2 Molecular analysis

**FIGURE 3:**
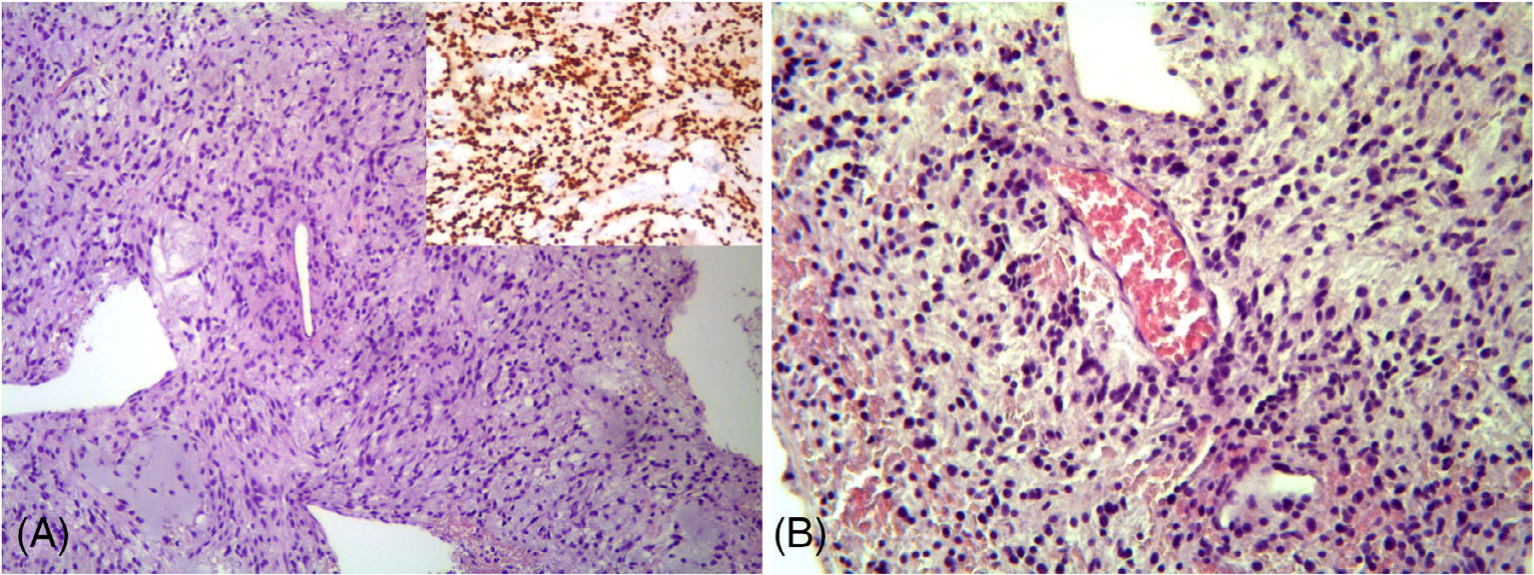
*Top panel*: Screenshot of the Alamut Visual software (Alamut® Visual—Interactive Biosoftware, Rouen, France) showing the results of *PTPN11* exon 13 target deep sequencing analysis. For each type of biological sample, the corresponding BAM alignments are displayed. The black arrows indicate the nucleotide change c.1520 C>A with the relative frequency for each sample. The Sanger sequencing electropherograms are also shown with the corresponding nucleotide change pointed by the red arrow. *Bottom panel*: Alamut Visual software screenshot showing *FGFR1* (NM_015850.3) exon 12. Red arrow and red square indicate the position 1632 in the coding sequence. The five Sanger sequencing electropherograms display the aforementioned position in five different tissues. Only the tumor sample shows the mutation c.1632C>A; p.(Asn544Lys)

No pathogenic variants in the PI3K/AKT/mTOR signaling pathway were detected on DNA isolated from skin biopsy. We then performed target deep sequencing with the RASopathy panel which revealed the pathogenic variant c.1520 C>A; p.(Thr507Lys) in *PTPN11* (NM_002834.3) with a variant allele frequency (VAF) of 12% (Figure 3). Therefore, a further target deep sequencing analysis with the same gene panel was performed on genomic DNA purified from FFPE tumor tissue, buccal swab, and blood samples. The same deleterious c.1520 C>A variant in *PTPN11* was detected in the FFPE tumor tissue (with a VAF of 36%), and was nearly absent in buccal swab and blood DNA (<0.2%) and could not be distinguished from background sequencing errors. All target NGS results were confirmed by Sanger sequencing (Figure 3, top panel). The NGS cancer‐hotspot analysis on tumor DNA revealed a c.1632C>A, p.(Asn544Lys) pathogenic variant of the *FGFR1* gene (NM_001174063.2) with a VAF of 36%, confirmed by Sanger sequencing. By the same approach, this variant was not found in blood, buccal swab, and skin biopsy DNA (Figure 3, bottom panel). Since the *FGFR1* variant was tested on blood, buccal swab, and skin biopsy only with Sanger sequencing it was not possible to exclude low levels of mosaicism.

## 4 DISCUSSION

The RASopathies are a group of phenotypically overlapping and genetically heterogeneous syndromes caused by germline mutations in genes encoding for proteins and regulators of the RAS/MAPK cascade, a key player signal pathway involved in cell growth and differentiation. Such mutations lead to an oversignaling in the RAS/MAPK cascade. The syndromes included within the RASopathies share a common clinical presentation with facial dysmorphisms, neurodevelopmental delay, stunted growth, cardiac malformations, and skeletal anomalies. NS (MIM #163950) is the most common of the RASopathies and *PTPN11* is the most frequently mutated among the genes of the RAS/MAPK signal cascade. Most germline activating variants of *PTPN11* typically lead to a NS phenotype.^8^

Mosaic RASopathies represent a distinct category of diseases and are caused by somatic pathogenetic variants in genes of the RAS/MAPK signaling pathway. Mosaic RASopathies are characterized by a wide spectrum of tissue segmental overgrowth/dysplasia including most often the eye, skin, heart, brain, bone, and soft tissues. Both segmental overgrowth and vascular malformations have been described in mosaic RASopathies.^5^, ^12^ The different disease entities belonging to the mosaic RASopathies include oculoectodermal syndrome (OMIM #600268), encephalo‐cranio‐cutaneous lipomatosis (OMIM #613001), Schimmelpenning–Feuerstein–Mims syndrome (OMIM #163200), cutaneous‐skeletal hypophosphatemia, RAS‐associated autoimmune lymphoproliferative syndrome (OMIM #614470). Somatic mutations in some genes of the RAS/MAPK pathway have also been associated with isolated malformations such as sebaceous and epidermal nevus (OMIM #162900), low‐flow vascular and arteriovenous malformations (OMIM #108010), melorheostosis (OMIM #155950), and congenital melanocytic nevus syndrome (OMIM #137550).

Somatic mutations in genes of the RAS/MAPK pathway are also a common finding in many types of cancers.^13^ The spectrum of pathogenic variants in these genes differ, in human cancer and in mosaic RASopathies, from the spectrum observed in germline RASopathies, suggesting the possibility of a germline lethality of the variants with higher functional impact and partially explaining the markedly different phenotype of mosaic RASopathies when compared to inherited forms. So far, mosaic RASopathies have been linked with only some mutations in some of the genes of the RAS/MAPK cascade, namely *BRAF*, *FGFR1*, *HRAS*, *KRAS*, *MAP2K1*, *NF1*, *NRAS*, and *RASA1*.^14^

Here, we report the first case of a mosaic RASopathy caused by a pathogenic variant in *PTPN11*, with a phenotype consistent with overgrowth and vascular proliferation associated with cancer predisposition and lacking any of the key features observed in the germline RASopathies. The phenotype observed in our patient more specifically resembled Klippel‐Trenaunay syndrome/diffuse capillary malformation with overgrowth, both disease entities included within the (PIK3CA)‐related overgrowth spectrum (PROS).^7^, ^9^

Germline gain‐of‐function variants in *PTPN11* have been typically associated with NS, and heterozygous variants with a dominant‐negative effect in NS with multiple lentigines (MIM #151100). Somatic *PTPN11* variants are associated with cancer, and frequently found inJMML.^8^ More recently, postzygotic mosaicism of a specific *PTPN11* variant, p.(Gln175His), has been linked to metachondromatosis.^15^ The p.(Thr507Lys) variant we found in our patient has been previously described as a somatic event in different types of cancers,^16^ and as germline event in two severely malformed fetuses^17^, ^18^ but not in live RASopathy patients, indicating the likely lethality of this variant in the germinal state.

The observation of a mosaic *PTPN11* pathogenic variant in a patient with segmental overgrowth overlapping the PROS phenotype is intriguing since both RAS–ERK (extracellular signal‐regulated kinase) and PI3K‐mTOR signaling pathways are crucial control mechanisms of cellular survival, differentiation, and proliferation in response to extracellular stimuli.^19^ Several hints suggest that these two cascades are connected in a complex cross‐talk network and intersect regulating their respective downstream functions. Recent reports evidenced a significant overlap between developmental disorders caused by gain‐of‐function variants of the RAS‐MAPK and the PI3K/AKT/mTOR pathways.^19^, ^20^

In PROS and some of the LOs, molecular testing is paramount as several actionable mutations identify candidates for a gene/pathway‐specific precision medicine approach based on inhibitors usage. At present, however, nearly 40% of patients with PROS‐like phenotypes have no mutation in genes of the PI3K/AKT/mTOR pathway.^7^ The finding of a variant in *PTPN11* causing a PROS‐like phenotype is noteworthy since it further supports that testing for somatic variants in RAS/MAPK pathway genes may reduce the fraction of cases with no molecular diagnosis and excluded from specific trials or compassionate use prescription. The somatic oncogenic variant (COSM13036) we identified in the *PTPN11* gene is potentially actionable as treatment with MEK‐inhibitors could be envisioned. Actually, response to MEK‐inhibition was observed in patients with pediatric low grade gliomas with RAS/MAPK/pathway activation^21^ as well as in complicated NS patients,^22^ suggesting this strategy could be promising. Theoretically, the case here presented could benefit the most from such treatment, being carrier of two mutations in this cascade, with the somatic *PTPN11* variant affecting part of the body—including tumor tissue—and a variant in *FGFR1* detected only in tumor tissue. MEK‐inhibition actually might result in improvements in both the body overgrowth phenotype and cancer proliferation control.

Somatic pathogenic variants in *FGFR1*—encoding the fibroblast growth factor receptor 1—in the embryo are responsible for another disorder belonging to mosaic RASopathies, namely a congenital overgrowth‐malformation phenotype known as encephalocraniocutaneous lipomatosis characterized by congenital anomalies of skin, scalp, eye, choristomas, and brain lipomas (ECCL, OMIM # 613001). Moreover, *FGFR1* is frequently mutated in multiple low‐grade neuroepithelial tumors including pilocytic astrocytoma,^23^ and patients with ECCL display an increased risk of pilocytic astrocytomas. Furthermore, *FGFR1* mutations in cancer tissues have been associated with spontaneous intratumoral hemorrhage, as observed in our patient.^24^ Key phosphorylation events in the RAS/MAPK cascade (e.g., in *PTPN11*) have been identified as potential switches mediating oncogenic pathway activation in glioblastomas.^25^ In the reported patient, the tumor tissue had a *PTPN11* VAF higher than that observed in noncancerous cutaneous overgrowth tissue, suggesting a clonal selection induced by the overactivation of the RAS/MAPK cascade. Oversignaling through this cascade is therefore enhanced by both the *PTPN11* and the *FGFR1* variant, likely representing two hits in the tumor progression. Given that the VAF of both the *FGFR1* and *PTPN11* variants were nearly identical in the tumor tissue suggesting that the variants co‐exist in the tumor cells, it is possible that the *FGFR1* mutation that initiated the clonal expansion and tumor progression. Given the mosaic nature of the variant and the variability in tissue sampling, it is possible that the difference in VAF is due to greater enrichment of affected cells in the tumor tissue sample than in the skin biopsy sample. These findings add novel insights into the interconnectedness of RAS/MAPK pathway mutations with cancer, and the still debated cancer predisposition of patients diagnosed with RASopathies and, specifically, *PTPN11* variants.^26^ Indeed, several reports suggest that patients with NS might carry a predisposition to develop glial or glioneuronal tumors brain tumors.^27^, ^28^ Further, in such cases, overactivation of the PI3K/AKT/mTOR pathway was demonstrated, consistent with a response to rapamycin analogues treatment, further supporting the existence of a strict interconnection of this pathway with the RAS/MAPK cascade.^27^ In addition, paralleling several observations in patients affected by PROS,^29^ cases with Wilms tumor (WT) have been described in patients with mosaic RASopathies,^5^, ^30^ and ultrasound screening for WT in both conditions has been suggested until the natural history is fully elucidated in larger cohort studies.^29^

In conclusion we report the first case of a mosaic RASopathy caused by a pathogenic variant in *PTPN11*, consistent with an overgrowth‐vascular malformation with cancer predisposition. This case further adds insights into the still largely not fully elucidated genetic bases of LOs. Moreover, clinically overlapping PROS, suggests that cases negative to molecular testing for mutations in the PIK3/AKT/mTOR genes should further be analyzed for variants in the RAS/MAPK cascade genes. These observations are relevant especially with a view of a precision medicine approach to overgrowth and related complications, including cancer.

## FUNDING INFORMATION

This research was partially funded by the Patients' Association “Associazione Nazionale Sindrome di Noonan e RASopatie ODV.”

## CONFLICT OF INTEREST

The authors declare no conflict of interest.

## Data Availability

The data that support the findings of this study are available on request from the corresponding author. The data are not publicly available due to privacy or ethical restrictions.
